# Diagnostic approach in a patient with Creutzfeldt-Jakob disease

**DOI:** 10.1590/1980-5764-DN-2021-0110

**Published:** 2022-05-23

**Authors:** José Wagner Leonel Tavares-Júnior, Renata de Oliveira Carvalho, Raul Raposo Pereira Feitosa, Flávia de Paiva Santos Rolim, Felipe Araújo Rocha, Milena Sales Pitombeira, George Linard Silva Malveira, João José Freitas de Carvalho, Norberto Anizio Ferreira Frota, Daniel Aguiar Dias

**Affiliations:** 1 Universidade Federal do Ceará, Departamento de Clínica Médica, Fortaleza, CE, Brazil.; 2 Hospital Monte Klinikum, Fortaleza, CE, Brazil.; 3 Hospital Geral de Fortaleza, Serviço de Neurologia, Fortaleza, CE, Brazil.; 4 Universidade de Fortaleza, Serviço de Neurologia, Fortaleza, CE, Brazil.; 5 Universidade Federal do Ceará, Departamento de Radiologia, Fortaleza, CE, Brazil.

**Keywords:** Prion Diseases, Creutzfeldt-Jakob Syndrome, Dementia, Doenças Priônicas, Síndrome de Creutzfeldt-Jakob, Demência

## Abstract

Prion diseases are an important cause of rapidly progressive dementias. Among them, the most common is sporadic Creutzfeldt-Jakob disease (CJD). It is a rare and incurable disease, with rapid progression to death.

**Objective:**

To describe the diagnostic approach of a patient with Creutzfeldt-Jakob disease.

**Methods:**

The diagnosis is established through the clinical picture associated with characteristic changes in the brain magnetic resonance imaging, the electroencephalogram, and analysis of specific changes in the cerebrospinal fluid.

**Results:**

The present report describes the case of a 53-year-old patient in the city of Fortaleza-CE. The diagnosis was made based on the clinical condition and through diagnostic tests, including 14-3-3 protein and RT QUIC analysis. Differential diagnosis was performed with other rapidly progressive causes, such as infectious and immune-mediated diseases.

**Conclusions:**

The diagnosis of probable sporadic CJD was established.

## RESUMO

As doenças priônicas são uma importante causa de demências rapidamente progressivas. Entre elas, a mais comum é a doença de Creutzfeldt-Jakob (DCJ) esporádica. É uma enfermidade rara e incurável, com rápida progressão para óbito.

**Objetivo:**

Descrever a abordagem diagnóstica de uma paciente com doença de Creutzfeldt-Jakob.

**Métodos:**

O diagnóstico é estabelecido pelo quadro clínico associado a alterações características na ressonância magnética cerebral, no eletroencefalograma e pela análise de alterações específicas no líquido cefalorraquidiano.

**Resultados:**

O presente relato descreve o caso de um paciente de 53 anos na cidade de Fortaleza (CE). O diagnóstico foi feito com base na condição clínica e por meio de testes diagnósticos, incluindo proteína 14-3-3 e análise *Real-Time Quaking-Induced Conversion* (RT QUIC). O diagnóstico diferencial foi realizado com outras causas rapidamente progressivas, como doenças infecciosas e imunomediadas.

**Conclusões:**

Por fim, foi estabelecido o diagnóstico de provável DCJ esporádica.

## INTRODUCTION

Creutzfeldt-Jakob disease (CJD) is the prototype of prion diseases, one of the main causes of rapidly progressive dementia (RPD). It is an incurable disease. CJD is subdivided into sporadic, familial, variant, and iatrogenic subtypes^1^. The sporadic subtype is the most common. It was first described in 1920 by Hans Creutzfeldt and later in 1921 and 1923 by Alfons Jakob^2,3^. Etiologically, it is caused by an agent called a prion, which was recognized in 1960 by Stanley Prusiner, which later gave him the Nobel Prize^4^. The disease-causing prion protein undergoes a conformational change, which in turn causes a change in the previously normal cellular prion protein^5^. Pathological changes in the disease include vacuolar lesions that give a spongiform appearance to the brain, preferably in the basal ganglia, thalamus, cerebellum, and cerebral cortex^6^.

## CASE REPORT

**Figure 1:**
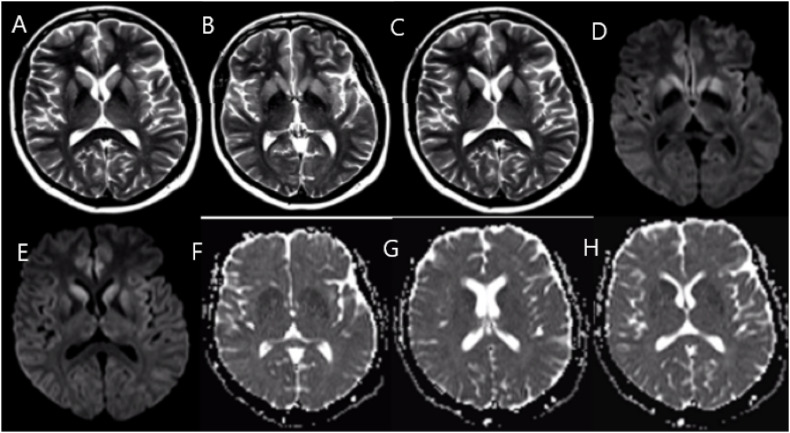
Brain magnetic resonance imaging scans demonstrating hypersignal in bilateral basal ganglia, insula, and anterior cingulate gyrus in T2 sequence (A, B, C) and hypersignal in the same regions in the diffusion sequence (D, E) with corresponding low signal in the ADC map (F, G, H), characterizing a true restriction to water molecules.

**Figure 2:**
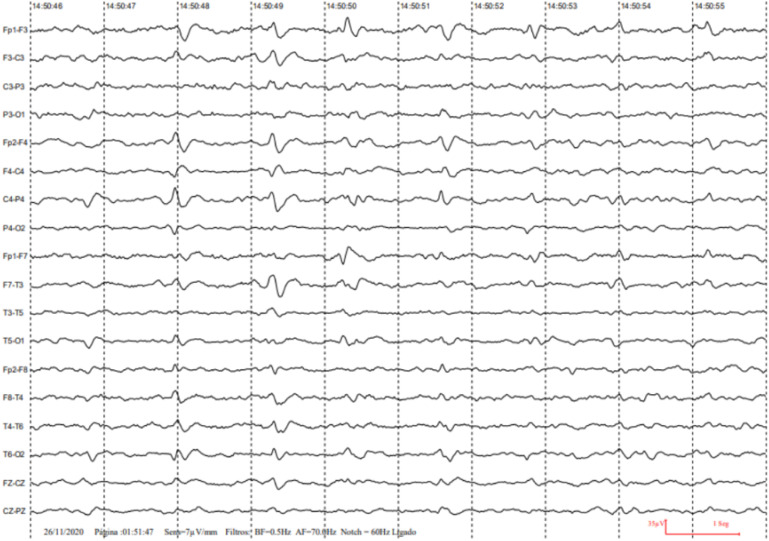
Periodic discharges on electroencephalogram.

A previously healthy 53-year-old patient started to complain of asthenia, fatigue, and anxiety, with excessive fear about contracting coronavirus. In the first week after the onset of symptoms, he started to present mental confusion with forgetfulness for recent facts, imbalance, and a few episodes of falls. Within 2 weeks of the onset of symptoms, she was evaluated by a neurologist, who found mild gait ataxia and suggested for cranial magnetic resonance imaging (MRI), in addition to brain arterial and venous angio MRI and electroencephalogram (EEG) video. The angio MRI showed no changes and the EEG video revealed nonspecific encephalopathy, whereas the MRI showed hypersignal in the diffusion, along with restriction, bilateral in insula, basal ganglia, and anterior cingulate (Figure 1). In addition to neuroimaging, she performed laboratory tests concurrently, which did not reveal abnormalities, but showed nonreactive VDRL, normal serum ammonia level, as well as normal renal, thyroid, and liver functions, in addition to negative serologies for HIV and hepatitis. After a few days, she worsened from mental confusion and imbalance, without new abnormalities observed under the neurological examination, and underwent a lumbar puncture. The partial analysis of the cerebrospinal fluid (CSF) revealed normal cells, glucose and protein levels, direct research and culture for normal pyogenic germs, fungi, and tuberculosis, as well as negative PCR for herpes virus types 1 and 2. In addition, CSF autoantibodies and 14-3-3 protein were requested. At that time, the hypothesis of RPD was formulated, with an emphasis on two major possibilities: prion disease or immune-mediated encephalitis. Given the delay in obtaining 14-3-3 protein, it was opted for hospitalization for pulse therapy with methylprednisolone 1 g/day for 5 days, followed by intravenous administration of immune globulin (IGIV) at a dose of 2 g/kg for 5 days. During hospitalization, MRI and EEG video were repeated without any change, and a worsening of the clinical picture was observed on memory and the appearance of spastic tetraparesis, hypophonia, and bradykinesia in the upper limbs and lower limbs, as well as optic ataxia and oculomotor apraxia. The evolution was rapid and, in 2 weeks of hospitalization, the patient was in akinetic mutism; therefore, gastrostomy (GTT) was performed due to dysphagia. She later developed fever due to aspiration pneumonia and was treated with antibiotics. She was discharged with GTT and akinetic mutism. After 30 days, she started to develop myoclonus and hence underwent the remaining examination, and the result revealed the presence of 14-3-3 protein in high titers. The family members were informed about the institution of palliative care, avoiding invasive measures. During evolution, the patient underwent three EEGs. In the first two EEGs, only a nonspecific slowing was revealed, while the third one, which was performed in November 2020, revealed bilateral generalized periodic activity (Figure 2). Subsequently, the patient underwent a lumbar puncture for RT QUIC analysis of the CSF which showed a positive result.

## DISCUSSION

The annual incidence of CJD is about 1 case per million people^7^. It is, therefore, a rare and incurable disease. The rarity of this condition is evidenced in different series of services specialized in dementia, when specifically evaluating the etiologies of RPD^8^. Sporadic CJD is the main form of CJD and is characterized by cognitive, visual, cerebellar, and motor (pyramidal/extrapyramidal) signs and symptoms, which are part of the existing diagnostic criteria for the disease^9^.

In addition to the clinical picture, some complementary examinations help in the diagnosis of the disease. The brain MRI shows lesions with hypersignal in the diffusion and FLAIR/T2 in the cerebral cortex and basal ganglia, like the patient in question^10^. The EEG, despite its low sensitivity, can demonstrate findings suggestive of triphasic waves or periodic complexes^11^. The CSF analysis can help in the research of proteins 14-3-3, tau, and p-tau that show an increase in the referred disease^12^. In addition, the detection of pathological prion protein in the nasal mucosa or CSF using an amplification technique reveals a high specificity and this analysis is called RT QUIC^13^.

The present case study demonstrates the importance of an appropriate investigation in situations of RPD^14^. This investigation involves the search of autoimmune encephalitis, central nervous system (CNS) infections, and metabolic and demyelinating conditions^15^. In our patient, clinical course after immunotherapy, CSF examination (including RT QUIC), laboratory tests, systemic neoplasms research, and brain MRI confirmed CJD, in addition to ruling out CNS infections, metabolic diseases, autoimmune, and paraneoplastic encephalitis. The possibility of performing RT QUIC in suspected cases is extremely important, given its high specificity in prion disease diagnosis^13^. In addition, investigation of autoimmune and paraneoplastic encephalitis and CNS infections in CSF is essential^14^.

In summary, patient in this report presented a typical clinical picture, corroborated by a typical MRI and EEG, and also high titers of 14-3-3 protein in the CSF, in addition to positive RT QUIC. The present case was investigated in the context of a COVID-19 pandemic, which raised concerns about hospitalization by the family. The presence of a health care plan by the patient made it possible for the family to carry out the tests quickly. However, even with the rapidity, the delay in the results of tests and the financial impossibility in the performance of autoantibodies in the CSF imposed the empirical treatment for encephalitis immune-mediated with MPIV and IGIV without improvement. Despite difficulties and limitations in carrying out elective tests imposed by the pandemic in the city of Fortaleza (hospitalization occurred at the peak of the pandemic in May 2020 in the city of Fortaleza), there was a rapidity in the formulation of the diagnostic hypothesis, performance of examinations, and hospitalization (from the first visit to the completion of the IGIV, it took exactly 20 days). The report of the present case helps keep the memory of this diagnosis alive in clinical practice, emphasizes the logistical difficulties faced in carrying out the most detailed CSF examinations even in the context of private medicine, and demonstrates the importance of defining the diagnosis of the cause of RPD for better acceptance of the diagnosis and end-of-life programming by the family.
